# Association Between Vericiguat and Clinical Outcomes Across Patients With Heart Failure: A Systematic Review and Meta-Analysis of Randomized Controlled Trials

**DOI:** 10.31083/RCM49095

**Published:** 2026-04-17

**Authors:** Yusra Minahil Nasir, Juveriya Yasmeen, Nishat Shama, Song Peng Ang, Neel Ajit Doshi, Novonil Deb, Haris Hamsakutty, Shamna Haris, Vikash Jaiswal, Jishanth Mattumpuram

**Affiliations:** ^1^Department of Internal Medicine, University of Oklahoma Health Sciences Centre, Oklahoma City, OK 73104, USA; ^2^Department of Internal Medicine, Saint Joseph Hospital, Chicago, IL 60657, USA; ^3^Department of Internal Medicine, Henry Ford Health, Detroit, MI 48202, USA; ^4^Division of Cardiology, Sarver Heart Center, University of Arizona, Tucson, AZ 85179, USA; ^5^Department of Internal Medicine, Mobile Infirmary Medical Center, Mobile, AL 36607, USA; ^6^Department of Internal Medicine, North Bengal Medical College and Hospital, 734012 Siliguri, India; ^7^Department of Cardiology, St. Mary Hospital, Kankakee, IL 60901, USA; ^8^Department of Cardiology Research, Larkin Community Hospital, South Miami, FL 33143, USA; ^9^Division of Cardiology, University of Louisville School of Medicine, Louisville, KY 40202, USA

**Keywords:** heart failure, vericiguat, mortality, prevention

## Abstract

**Background:**

Heart failure (HF) remains a major global health burden, with mortality continuing to rise despite therapeutic advances. Vericiguat, a soluble guanylate cyclase stimulator, has demonstrated potential benefit in patients with worsening HF with reduced ejection fraction (HFrEF), although results across randomized trials have been inconsistent.

**Methods:**

We conducted a systematic literature search across PubMed, Scopus, and ClinicalTrials.gov for relevant articles from inception through September 30th, 2025. Outcomes were reported as pooled odds ratios (ORs) with corresponding 95% confidence intervals (CIs). Statistical significance was defined as a 95% confidence interval not crossing 1.0 with a two-tailed *p*-value < 0.05.

**Results:**

Five randomized controlled trials (RCTs) with 12,877 patients (6857 in the vericiguat group and 6020 in the placebo group) were included. Vericiguat demonstrated a borderline but non-significant reduction in composite outcome of cardiovascular death (CVD) or hospitalization for HF (OR 0.92, 95% CI 0.85–1.00; *p* = 0.05), hospitalization for HF (OR 0.93, 95% CI 0.85–1.02; *p* = 0.14), and all-cause mortality (ACM) (OR 0.91, 95% CI 0.81–1.01; *p* = 0.07).

**Conclusion:**

The findings of this study suggest that Vericiguat, when added to guideline-directed medical therapy in patients with heart failure, was associated with a borderline, non-significant reduction in the risk of the composite outcome of cardiovascular death or heart failure hospitalization, as well as all-cause mortality. Further large-scale randomized trials are warranted to better define its clinical benefit.

## 1. Introduction

According to the Global Burden of Disease 2021 data, the global age-standardized prevalence of heart failure (HF) was estimated at approximately 148.1 cases per 100,000 population [1]. In recent years, several guideline-directed medical therapies have been approved for the treatment of HF; however, contemporary studies continue to demonstrate an alarming rise in HF-associated mortality in Europe and the United States [2, 3].

Vericiguat, a soluble guanylate cyclase stimulator, has been proposed to improve myocardial and vascular function, thereby mitigating adverse HF outcomes. Current guidelines from the American College of Cardiology (ACC) and the American Heart Association (AHA) place vericiguat in Class IIb recommendations as an adjunct therapy for patients with worsening heart failure with reduced ejection fraction (HFrEF) [4]. A recently published VICTOR trial demonstrated that vericiguat, when added to standard therapy, was associated with a reduction in composite outcomes [5]. However, findings across prior randomized trials have been inconsistent. Therefore, we aimed to perform a pooled analysis of randomized controlled trials to evaluate the efficacy of vericiguat in patients with HF.

## 2. Materials and Methods

This meta-analysis was conducted and reported following the PRISMA (Preferred Reporting Items for Systematic Reviews and Meta-analysis) 2020 guidelines and performed according to established methods, as described previously [6, 7]. We conducted a systematic literature search in PubMed, Scopus, and ClinicalTrials.gov using predefined MESH terms by using “AND” and “OR”. The search strategy included: (“vericiguat”[MeSH Terms] OR “vericiguat”[Title/Abstract] OR “Verquvo”[Title/Abstract] OR “BAY 1021189”[Title/Abstract] OR “BAY1021189”[Title/Abstract] OR “MK-1242”[Title/Abstract] OR “MK1242”[Title/Abstract]) AND (“heart failure”[MeSH Terms] OR “heart failure”[Title/Abstract] OR “cardiac failure”[Title/Abstract] OR “congestive heart failure”[Title/Abstract] OR “CHF”[Title/Abstract] OR “heart decompensation”[Title/Abstract] OR “myocardial failure”[Title/Abstract] OR “HFrEF”[Title/Abstract] OR “HFpEF”[Title/Abstract] OR “reduced ejection fraction”[Title/Abstract] OR “preserved ejection fraction”[Title/Abstract]).

This study aimed to include all available RCTs published up to September 2025 that compare the effects of vericiguat with placebo in patients with heart failure and reported relevant outcomes of interest. Non-randomized or non-clinical studies, studies not involving patients with heart failure or use of vericiguat, trials without a control group, and those lacking extractable clinical outcomes were excluded.

We performed a conventional meta-analysis for the outcomes and applied the DerSimonian and Laird random-effects model to account for study heterogeneity. Outcomes were reported as pooled odds ratios (ORs) and their corresponding 95% confidence intervals (CIs). Statistical significance was defined as a 95% confidence interval not crossing 1.0 with a two-tailed *p*-value < 0.05. All the analyses were conducted using STATA version 17.1 (StataCorp, College Station, TX, USA). This study was not prospectively registered in a systematic review registry (e.g., PROSPERO).

## 3. Results

The initial search strategy yielded 425 articles, of which 79 duplicates were removed, and 331 were excluded after title and abstract screening.

**Fig. 1.**
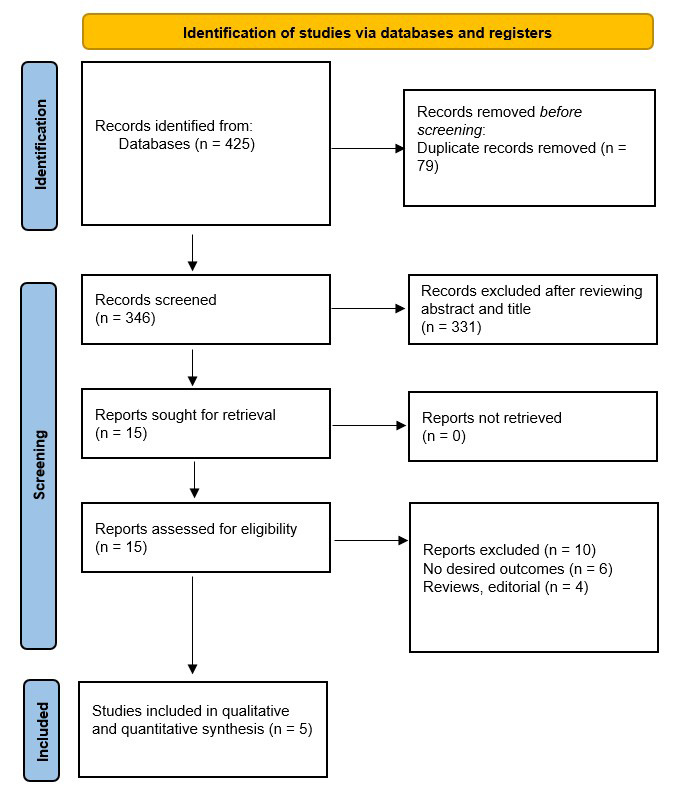
**Prisma flow chart of included studies**.

A total of 5 randomized controlled trials with 12,877 patients (6857 in the vericiguat group and 6020 in the placebo group) were included in the analysis (Fig. 1) [5, 8, 9, 10, 11]. Baseline characteristics of the patients in the included studies are given in Table 1.

**Table 1.** **Baseline demographics, comorbidities, and study characteristics of studies included in the meta-analysis**.

Trials	Group	Sample	Ejection Fraction	Age, years	Male, n	Diabetes, n	NT-proBNP (pg/mL), median	BMI (kg/m^2^)	Follow up
VICTOR, 2025	Vericiguat	3053	<45	68	2326	1274	-	27.5	18.5
	Placebo	3052	<45	68	2339	1311	-	27.7	
VICTORIA, 2020	Vericiguat	2526	<45	65.7	1921	NR	NR	27.7	10.8 months
	Placebo	2524	<45	67.2	1921	NR	NR	27.9	
SOCRATES-PRESERVED, 2017	Vericiguat	384	>45	73.25	203	185	1275.5	30.2	12 Weeks
	Placebo	93	>45	74	47	47	975	30.1	
SOCRATES-REDUCED, 2015	Vericiguat	364	<45	68	293	178	2885	28.25	16 weeks
	Placebo	92	<45	67	73	41	4043	27	
VITALITY-HFpEF, 2020	Vericiguat	527	>45	72.65	263	235	1351.8	30.65	24 weeks
	Placebo	262	>45	72.8	141	123	1664.2	30.7	

NT-proBNP, N-terminal pro-BNP; BMI, body mass index.

**Fig. 2.**
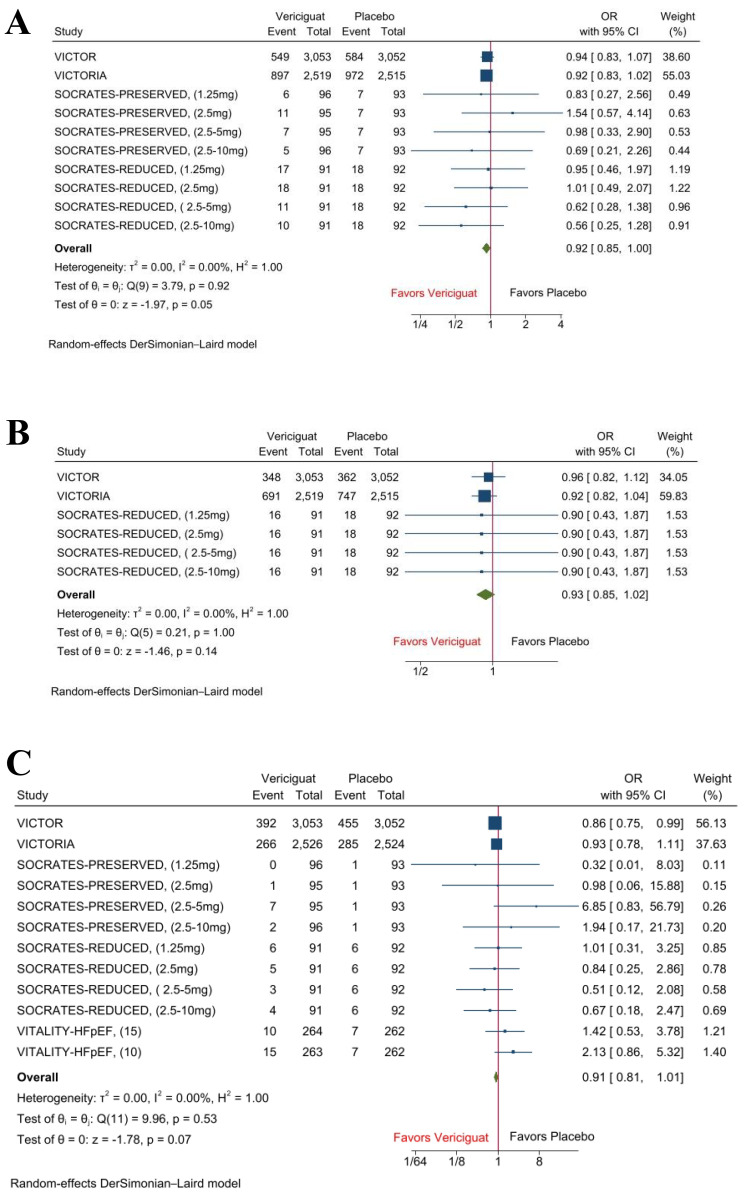
**Forest plots of composite outcome, hospitalization for heart failure, and all-cause mortality**. (A) Composite outcome of cardiovascular death or hospitalization for heart failure. (B) Hospitalization for heart failure. (C) All-cause Mortality.

Pooled analysis demonstrated that, compared with placebo, vericiguat-treated patients showed a borderline but non-significant reduction in the composite outcome of cardiovascular death or hospitalization for heart failure (OR 0.92 [95% CI: 0.85–1.00], *p* = 0.05, I^2^ = 0%). Similar non-significant trends were observed for reduction in hospitalization for heart failure (OR 0.93 [95% CI: 0.85–1.02], *p* = 0.14, I^2^ = 0%), and all-cause mortality (OR 0.91 [95% CI: 0.81–1.01], *p* = 0.07, I^2^ = 0%), (Fig. 2).

## 4. Discussion

Worsening HFrEF is characterized by impaired nitric oxide bioavailability, oxidative stress-mediated soluble guanylate cyclase (sGC) dysfunction, and consequent suppression of cGMP signaling. Vericiguat directly stimulates sGC and enhances its sensitivity to endogenous nitric oxide, thereby restoring cGMP production. Our study demonstrates that vericiguat, when added to guideline-directed therapy in HFrEF, was associated with a borderline but non-significant reduction in the composite outcome of CVD or HF hospitalization, as well as all-cause mortality. Although the endpoints did not reach statistical significance, the consistent direction of effect favoring vericiguat suggests a potentially favorable treatment effect. The absence of heterogeneity across outcomes (I^2^ = 0%) further strengthens the internal consistency of these findings.

For patients with HFrEF, these findings suggest that vericiguat can be considered as an add-on therapy following guideline-directed medical therapy, particularly in those with recent worsening (e.g., heart failure hospitalization or need for intravenous diuretics) and adequate blood pressure control. This is consistent with the 2022 AHA/ACC/HFSA guideline, which supports consideration of vericiguat in symptomatic HFrEF patients with recent decompensation despite optimal therapy (Class IIb), as well as the 2024 ACC Expert Consensus Decision Pathway, which positions vericiguat for high-risk patients already receiving optimal therapy. In clinical practice, vericiguat is administered once daily with food, typically initiated at 2.5 mg and uptitrated to 5 mg and 10 mg as tolerated, with close monitoring of blood pressure and hemoglobin.

Across individual trials, observed differences likely reflect variation in enrolled patient populations and background medical therapy. The VICTORIA trial, which enrolled patients shortly after a worsening heart failure event, demonstrated a statistically significant reduction in the composite outcome of cardiovascular death or heart failure hospitalization with vericiguat compared with placebo (*p* = 0.02), findings that are directionally consistent with our analysis [9]. In contrast, the VICTOR trial—conducted in ambulatory, well-treated patients with HFrEF without recent decompensation, was neutral for the composite outcome but suggested a reduction in cardiovascular mortality with vericiguat, indicating potential benefit even when effects on hospitalization were limited [5]. The pooled participant-level analysis of VICTORIA and VICTOR also demonstrated a consistent reduction in the composite outcome and suggested that patients with intermediate natriuretic peptide levels may derive the greatest benefit [12]. Early-phase and HFpEF studies (SOCRATES-PRESERVED and VITALITY-HFpEF) were neutral for clinical outcomes and were not designed to assess this composite endpoint, whereas SOCRATES-REDUCED demonstrated early signals of benefit in HFrEF and strengthened the rationale for further large-scale investigation [5, 10, 11].

A prior meta-analysis of randomized trials by Ma *et al*. [13] reported that vericiguat improved the composite of cardiovascular death or heart-failure hospitalization, while individual endpoints including hospitalization alone, cardiovascular death, all-cause mortality, and adverse events were not significantly different between groups. These findings remain consistent with our results, which demonstrate a borderline but non-significant reduction in composite and mortality outcomes. Safety outcomes across randomized trials were consistent with the established safety profile of vericiguat, with higher rates of hypotension and anemia observed and no new safety signals were identified. These findings support careful blood pressure guided titration, particularly early following clinical decompensation and in patients receiving concomitant vasodilator therapy.

Collectively, these findings suggest that vericiguat may be best utilized as a risk-targeted adjunct therapy in patients with HFrEF who remain symptomatic and at high residual risk following recent decompensation despite optimized guideline-directed medical therapy, rather than as a routine treatment for all patients with heart failure.

## 5. Limitations

This study has several limitations. First, it is a study-level meta-analysis and lacks individual patient-level data. Second, studies varied in patient population, follow-up periods, dose, and duration of treatment, which may introduce some bias. Finally, we included some phase II and phase III trials as well, which may also introduce bias in the main findings.

## 6. Conclusion

This study’s findings suggest that vericiguat, when added to guideline-directed therapy in patients with HF, was associated with a borderline but non-significant reduction in the risk of composite outcomes of cardiovascular death or heart failure hospitalization and all-cause mortality. Further large scale, adequately powered randomized trials are warranted to better define its clinical benefit. Future studies should focus on identifying patient subgroups most likely to derive clinical benefit from vericiguat.

## Availability of Data and Materials

All data reported in this paper will be shared by the first author upon request (Yusra Minahil Nasir, Yusra-nasir@ou.edu).

## Supplementary Material

Supplementary material associated with this article can be found, in the online version, at https://doi.org/10.31083/RCM49095.
